# Functional transition of CA2 pyramidal neurons along the proximodistal axis determines resonance frequency preference

**DOI:** 10.1038/s41598-026-39754-3

**Published:** 2026-02-17

**Authors:** Pia Kruse, Amelie Eichler, Katharina Brockmeyer, Maximilian Lenz

**Affiliations:** 1 Institute of Neuroanatomy and Cell Biology, Hannover Medical School, Carl-Neuberg-Straße 1, 30625 Hannover, Germany; 2 Institute of Neuroanatomy and Cell Biology, Hannover Medical School, Carl-Neuberg-Straße 1, 30625 Hannover, Germany

**Keywords:** CA2, Resonance, Frequency preference, Hippocampus, Biological techniques, Neuroscience

## Abstract

The hippocampal CA2 region has recently been linked to oscillatory activity, spatial navigation, and social behavior. Its predominant neuronal population, CA2 pyramidal neurons (CA2-PNs), critically contributes to hippocampal computations, yet the functional properties along the proximodistal extension of CA2 remain incompletely defined. In organotypic entorhino-hippocampal tissue cultures, we combined PCP4-based localization with single-cell reconstructions and whole-cell patch-clamp recordings to relate neuronal position to structure, synaptic transmission, and intrinsic properties in identified CA2-PNs. Dendritic morphology and spontaneous EPSCs did not correlate with position along the proximodistal axis. By contrast, intrinsic membrane properties, encompassing input resistance, rheobase and action potential metrics, varied gradually. Distal neurons exhibited a pronounced hyperpolarization-induced sag and subthreshold membrane resonance with a frequency preference shifting from delta frequency toward the lower theta range in a subset of cells along the proximodistal axis. Together, these findings reveal location-dependent frequency tuning in CA2 along the proximodistal axis and suggest that a gradient of intrinsic properties sets the conditions for input- and phase-specific recruitment of CA2 during hippocampal network activity.

**Supplementary Information:**

The online version contains supplementary material available at 10.1038/s41598-026-39754-3.

## Introduction

The hippocampal circuit is tightly linked to memory formation and recall. While the classical trisynaptic pathway of excitatory connections has primarily focused on the connectivity between dentate granule cells (dGCs) and pyramidal neurons (PNs) in CA3 and CA1 ^1–3^, the CA2 region has remained comparatively unexplored. Nevertheless, recent studies indicate a fundamental role of CA2 in spatial navigation and social behavior^4–6^, while the structural and functional cellular correlates of these processes require further investigation.

The boundaries of CA2 vary across studies and rely on cytoarchitecture, where initial studies noted a CA2-related ‘bump’ in the pyramidal cell layer of several species^7,8^. In addition to cytoarchitecture, recent studies revealed that CA2 can be reliably identified by distinct marker expression, such as RGS14 and PCP4 ^9,10^. Located between CA3 and CA1, CA2 is a highly connected area receiving specialized input from CA3 Schaffer collaterals, from layer II of the entorhinal cortex (EC), and a graded input from dGC mossy fibers that predominantly target proximal CA2-PNs^11^. Consistent with its connectivity, previous work described network motifs unique to CA2, such as strong feed-forward inhibition upon Schaffer collateral stimulation^12^. Whereas Schaffer collateral synapses exhibit limited plasticity, highly plastic and excitable inputs have been observed at distal portions of PN-dendrites^13–15^. Notably, substantial structural heterogeneity among CA2-PNs has long been recognized^16^. Proximodistal gradients in functional intrinsic and synaptic properties (including resting membrane potential, input resistance, sag, and after-hyperpolarization) have previously been described, indicating that CA2 is functionally organized along its proximodistal axis^17^. However, it remains insufficiently understood whether intrinsic frequency-selective properties, specifically subthreshold resonance and related tuning of membrane responsiveness, vary systematically across CA2 and how these features relate to cellular position. Moreover, *in vitro* preservation of position-dependent intrinsic tuning in common research models for the entorhino-hippocampal circuitry, which avoid acute axotomy inherent to acute slice preparations, remains widely unexplored.

In this study, we characterized structural and functional heterogeneity of CA2-PNs in organotypic entorhino-hippocampal tissue cultures. Using single-cell reconstructions and whole-cell patch-clamp recordings, we identified a proximodistal gradient of functional properties in CA2-PNs, while overall dendritic morphology remained comparable. Specifically, we observed a location-dependent frequency preference: distal CA2-PNs displayed resonance that gradually shifted from delta frequency toward the lower theta range, whereas proximal cells showed no detectable resonance. Given this spatially distinct frequency preference, our results suggest a location-dependent responsiveness to differential network activity patterns within the CA2 region.

## Results

### CA2 can be identified by PCP4 staining in organotypic entorhino-hippocampal tissue cultures

**Fig. 1:**
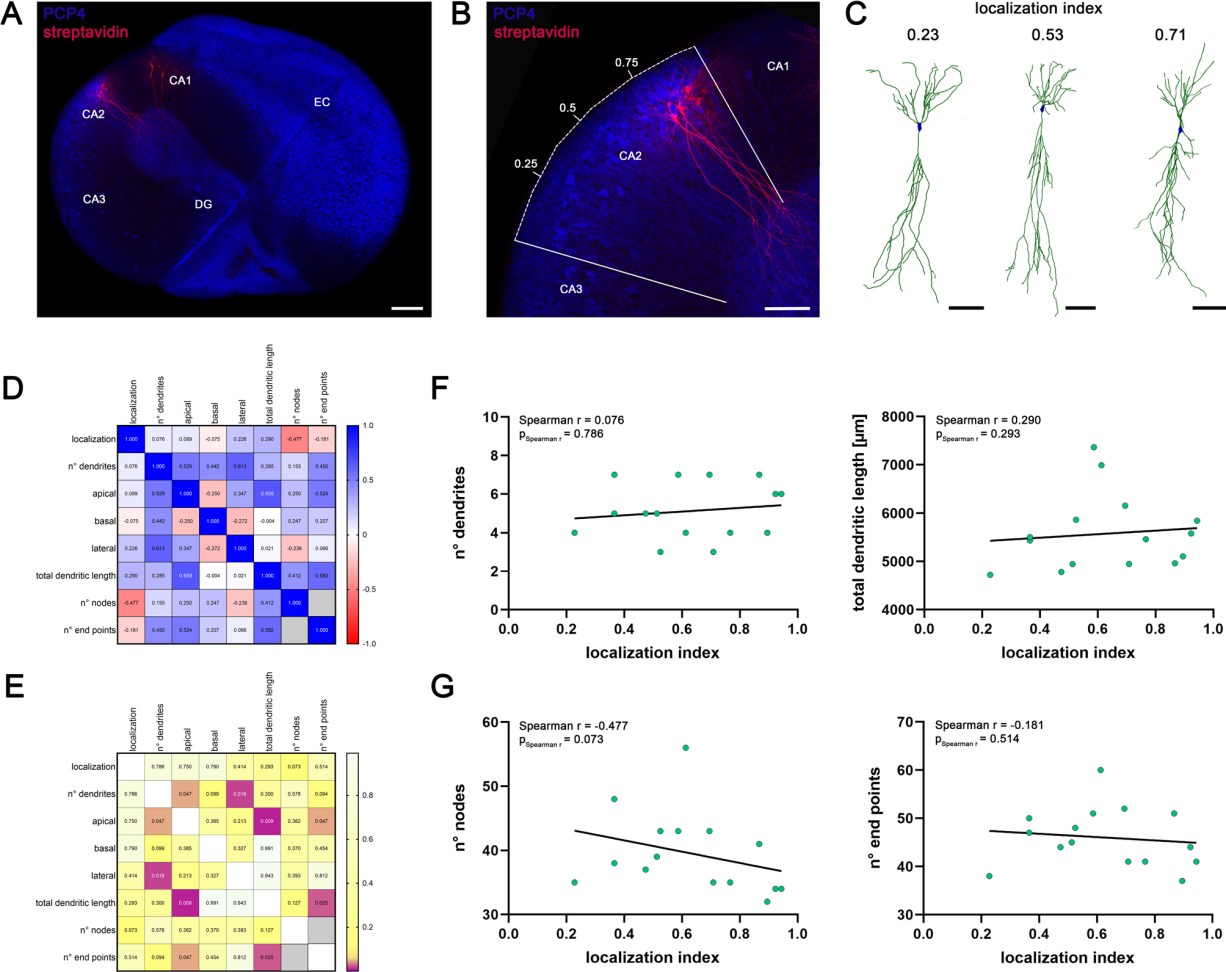
Dendritic morphology of CA2-PNs does not depend on location along the proximodistal axis. (**A**) Sample image of an organotypic entorhino-hippocampal tissue culture. PCP4 was used to identify the CA2 region. Note that pyramidal neurons in the CA2 and CA1 region were visualized post hoc by staining with streptavidin. DG, dentate gyrus; EC, entorhinal cortex; CA, cornu ammonis. Scalebar, 200 μm. (**B**) Close-up of the CA2 region from A identified by PCP4 staining. White lines indicate the border to the CA1 and CA3 region, respectively. Numbers represent the localization index as the relative location of neurons. Scalebar, 100 μm. (**C**) Sample images of reconstructed CA2 pyramidal neurons along the proximodistal axis. The respective localization index is reported above each cell. Scalebars, 100 μm. (**D**, **E**) Correlation matrix displaying Spearman correlations of continuous numerical data (mean values, **D**) and corresponding p-values (**E**). Significant results are indicated as violet squares (**E**; n = 15 CA2-PNs). Correlative analysis of values that inherently depend on each other (number of nodes and number of endpoints) were excluded from the correlation matrix and are indicated as gray squares. (**F**, **G**) Neither number of dendrites and total dendritic length (**F**) nor number of dendritic nodes and end points (**G**) significantly correlate with the CA2-PN location. Linear regression is indicated with a black line (p-values of Spearman r and Spearman r for correlation analysis are reported in the graphs). Individual values are indicated by colored dots. Source data are provided in Table S1.

We used organotypic entorhino-hippocampal slice cultures to investigate the structural and functional properties of CA2-PNs (Fig. 1). PCP4 immunofluorescence staining was used to delineate the CA2 region within the hippocampus (Fig. 1A, B). In addition to labeling CA2, PCP4 was also found in entorhinal regions and the dentate gyrus (Fig. 1A). We used whole-cell patch-clamp recordings with *post hoc* labeling of neurons, which enabled the localization of cells within the CA2 region (Fig. 1B). To determine comparable position metrics along the proximodistal axis across cultures, we computed a PCP4-based localization index, where 0.0 denotes the CA3 border and 1.0 the CA1 border. After calculating the cellular localization index, *post hoc* visualization enabled dendritic reconstructions of individual CA2-PNs (Fig. 1C). Together, this workflow relates CA2-PN position to their structural and functional properties.

### Structural properties of CA2-PNs do not correlate with their proximodistal location

In a next step, we correlated dendritic morphologies reconstructed from CA2-PNs with their localization index (Fig. 1D-G). A correlation matrix suggested no significant associations between the localization index and dendritic morphology metrics *in vitro* (Fig. 1D, E). Furthermore, location-dependent plots highlighted cellular heterogeneity across CA2 in key dendritic parameters, including number of dendrites, total dendritic length, number of nodes, and number of endpoints (Fig. 1F, G). Notably, significant correlations were observed in between morphological dendritic parameters, such as the presence of lateral dendrites (branching within the pyramidal cell layer) and the number of dendrites (Spearman’s *r* = 0.613, *p* = 0.018), between number of apical dendrites and total dendritic length (*r* = 0.659, *p* = 0.009), and between total dendritic length and number of endpoints (*r* = 0.582, *p* = 0.025; Fig. 1D, E). Although a limited number of cells were reconstructed, we conclude that dendritic morphological parameters of CA2-PNs do not show gross morphological changes along a proximodistal axis in organotypic tissue cultures.

### Spontaneous excitatory synaptic transmission does not correlate with CA2-PN location

**Fig. 2:**
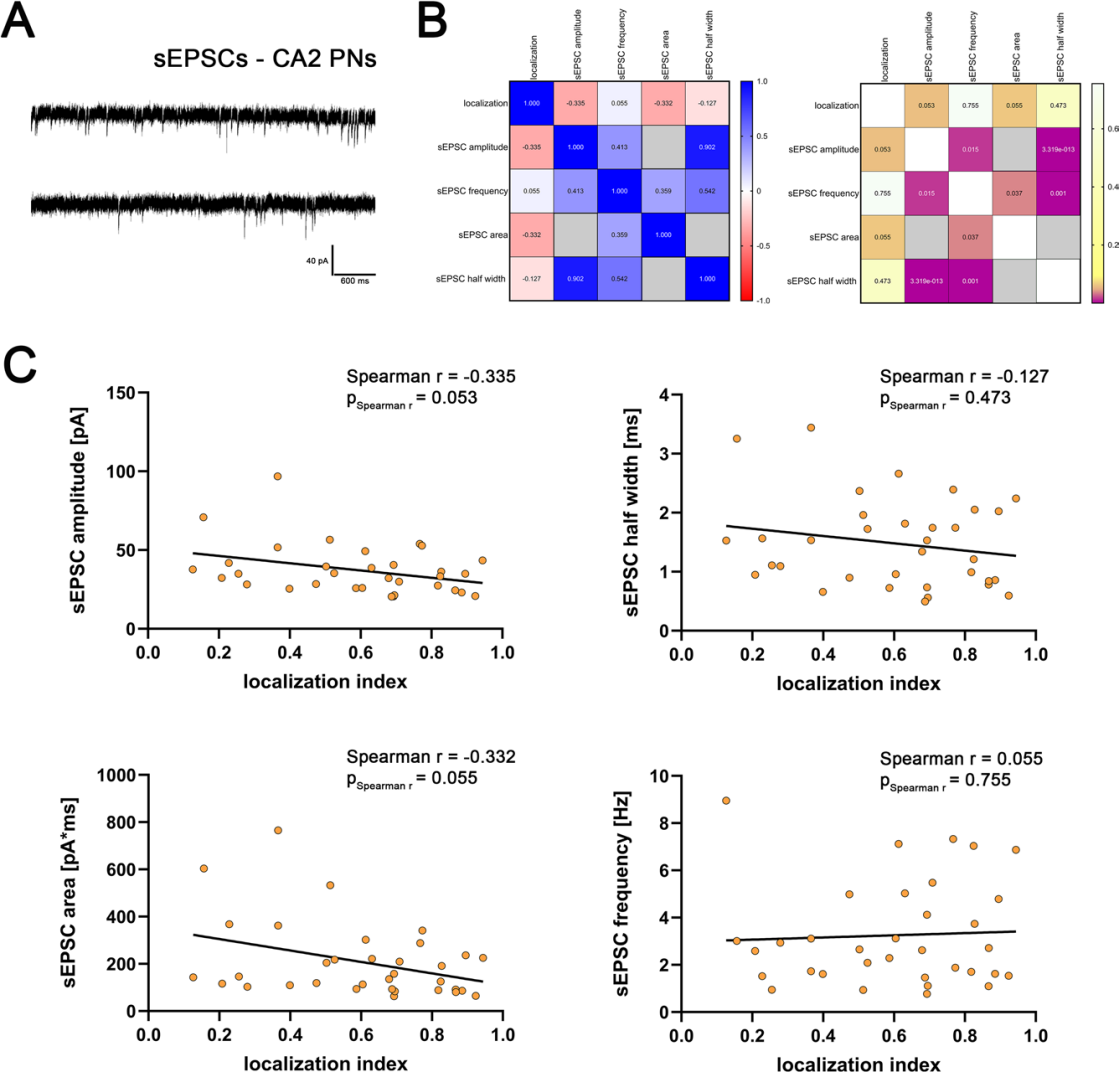
Synaptic parameters in sEPSCs of CA2-PNs show no correlation with neuronal location. (**A**) Sample traces of sEPSC recordings of CA2-PNs. (**B**) Correlation matrix displaying Spearman correlations of continuous numerical data (mean values, left) and corresponding p-values (right). Correlative analysis of values that inherently depend on each other (sEPSC area and sEPSC half width; sEPSC area and sEPSC amplitude) were excluded from the correlation matrix and are indicated as gray squares. Significant results are indicated as violet squares (n = 34 CA2-PNs). (**C**) Synaptic parameters such as sEPSC amplitude, half width, area and frequency do not significantly correlate with the location of the CA2-PNs along the proximodistal axis. Linear regression is indicated with a black line (p-values of Spearman r and Spearman r for correlation analysis are reported in the graphs). Individual values are indicated by colored dots. Source data are provided in Table S2.

Using whole-cell patch-clamp recordings, we assessed excitatory neurotransmission via spontaneous excitatory postsynaptic currents (sEPSCs) in CA2-PNs (Fig. 2A). The correlation matrix indicated substantial interdependence among sEPSC parameters, i.e., amplitude, half-width, area, and frequency (Fig. 2B). By contrast, none of these sEPSC parameters correlated significantly with the CA2-PN localization index (Fig. 2C; cf. Figure 2B). However, we noted a non-significant trend toward higher sEPSC amplitude and area in proximal CA2-PNs. We therefore conclude that sEPSC properties do not show a prominent proximodistal gradient within this data set.

### Input resistance and Sag ratio of CA2-PNs show a proximodistal gradual change

**Fig. 3:**
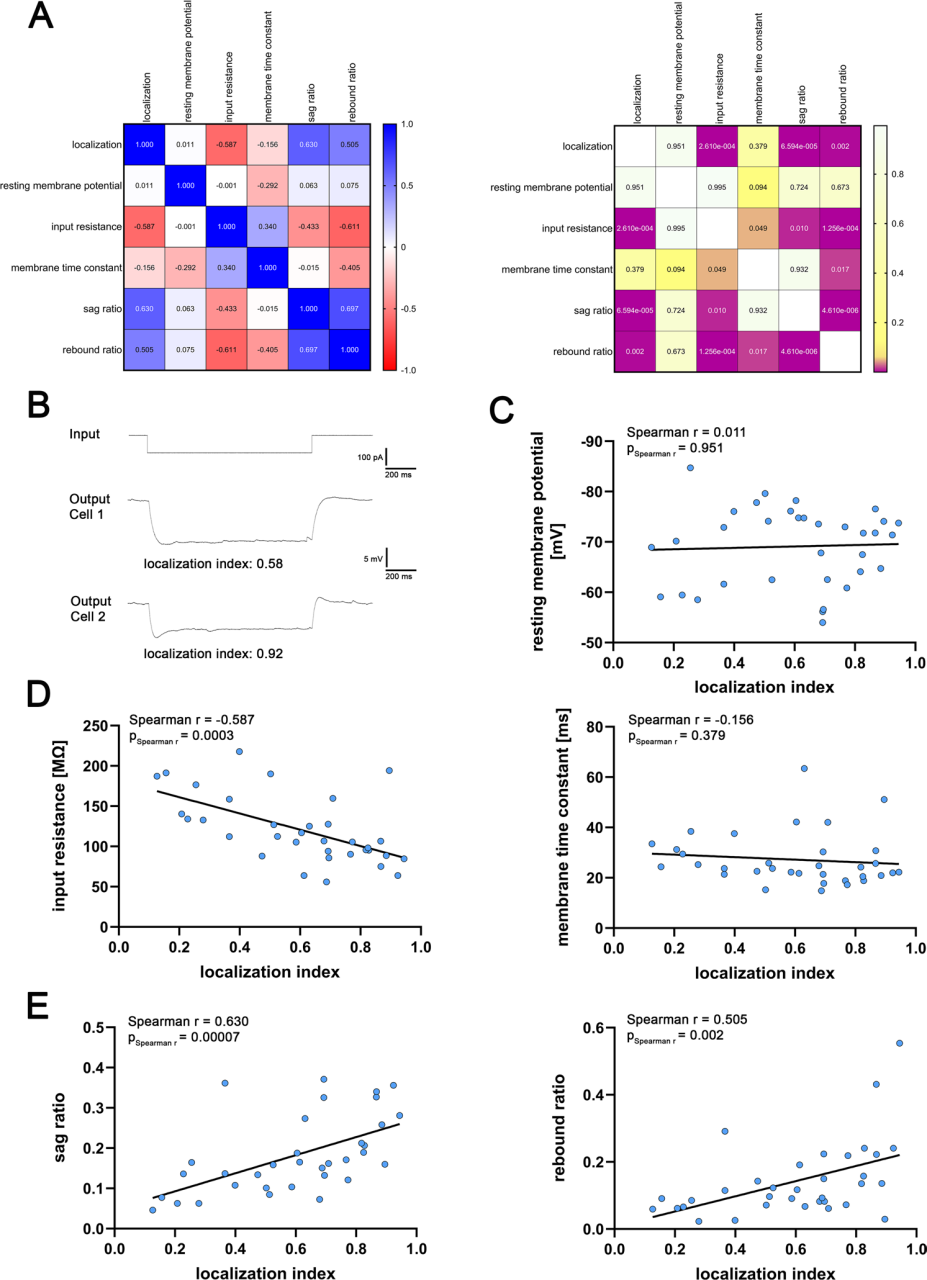
Intrinsic membrane properties of CA2-PNs dynamically change along the proximodistal axis. (**A**) Correlation matrix displaying Spearman correlations of continuous numerical data (mean values, left) and corresponding p-values (right). Significant results are indicated as violet squares (n = 34 CA2-PNs). (**B**) Voltage membrane response of two CA2-PNs (output, middle and lower trace) at a current injection of -100 pA (input, upper trace). The localization index of the cells is reported below the respective trace. (**C**)-(**E**) While resting membrane potential and membrane time constant do not correlate with the location of CA2-PNs, input resistance, sag ratio and rebound ratio significantly correlate with the location of CA2-PNs (p-values of Spearman r and Spearman r for correlation analysis are reported in the graphs). Individual values are indicated by colored dots. Source data are provided in Table S3.

Subsequently, we assessed passive membrane properties of CA2-PNs using IV-curve recordings (Fig. 3). Correlation matrix analysis indicated robust associations between the localization index and selected membrane properties, as well as interrelationships among intrinsic parameters (Fig. 3A). Compared to previous *ex vivo* studies^5,14,18^, resting membrane potentials were within a similar range, whereas CA2-PNs in organotypic tissue cultures displayed a higher apparent input resistance. Along the proximodistal axis, resting membrane potential did not correlate with CA2-PN location (Fig. 3C). By contrast, input resistance showed a significant correlation, with higher values near CA3 that progressively decreased towards CA1 (Fig. 3D, left). No correlations were observed for the membrane time constant (Fig. 3D, right). Based on the input resistance differences, we conclude that proximal CA2-PNs (near CA3) have distinguished passive membrane properties when compared to their distal counterparts. Additionally, we observed a positive correlation between the localization index and both sag ratio and rebound ratio, which is consistent with a gradual increase of a hyperpolarization-induced sag in distal CA2-PNs (Fig. 3E).

### Action potential properties of CA2-PNs vary along the proximodistal axis

**Fig. 4:**
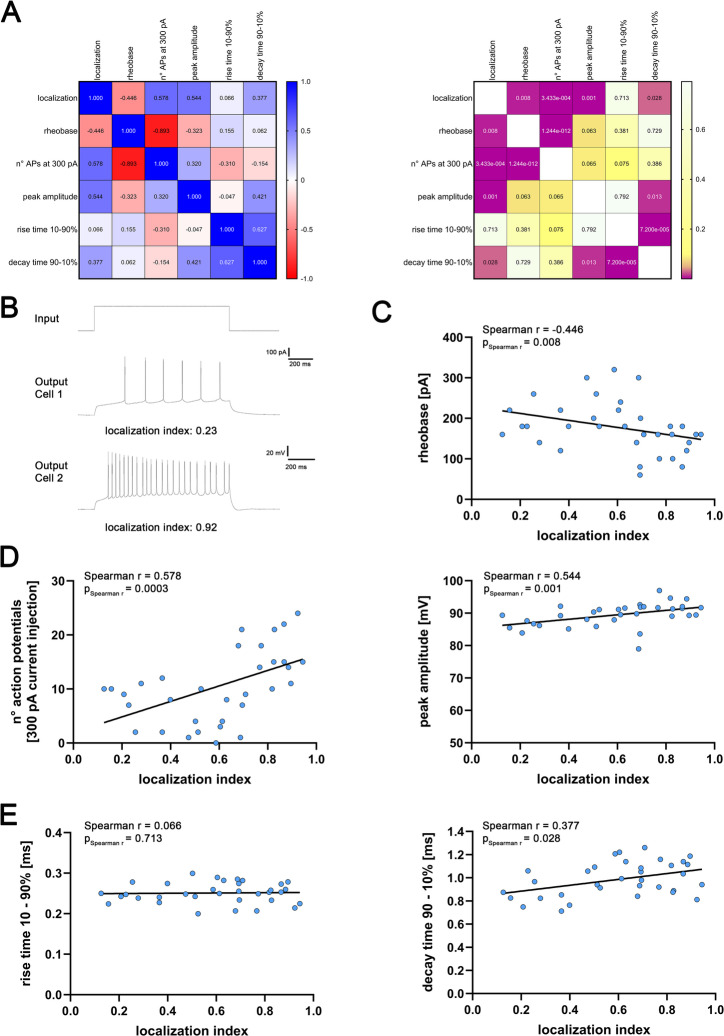
Active properties and action potential kinetics reveal a gradual change along the proximodistal axis. (**A**) Correlation matrix displaying Spearman correlations of continuous numerical data (mean values, left) and corresponding p-values (right). Significant results are indicated as violet squares (n = 34 CA2-PNs). (**B**) Voltage membrane response of two CA2-PNs (output, middle and lower trace) at a current injection of 300 pA (input, upper trace). The localization index of the cells is reported below the respective trace. (**C**)-(**E**) While rise time does not correlate with the location of CA2-PNs, rheobase, n° of action potentials at 300 pA current injection, peak amplitude and decay time of action potentials significantly correlate with the location of CA2-PNs (p-values of Spearman r and Spearman r for correlation analysis are reported in the graphs). Individual values are indicated by colored dots. Source data are provided in Table S4.

Next, we extended our analysis of intrinsic properties by quantifying action potential properties and firing output of CA2-PNs using depolarizing current injections (Fig. 4A, B). A correlation matrix indicated position-dependent associations between the localization index and selected action potential (AP)-related measures, while at the same time showing a strong interdependency of active membrane properties (Fig. 4A). Rheobase showed a negative correlation with the localization index, indicating that the current required to elicit the first spike was lower toward the CA1 border (Fig. 4C). Consistent with this, for identical current injections, firing rates correlated positively with the localization index, i.e., CA2-PNs closer to CA1 fired at higher frequencies (Fig. 4D, left). Moreover, AP peak amplitude increased toward CA1 (Fig. 4D, right). While AP rise time did not correlate with CA2-PN position, AP decay time showed a positive correlation with the localization index (Fig. 4E). Together, these data and reveal a proximodistal gradient in action potential properties within CA2 *in vitro*.

### Distal CA2-PNs display resonance features partially reaching theta-band frequencies

**Fig. 5:**
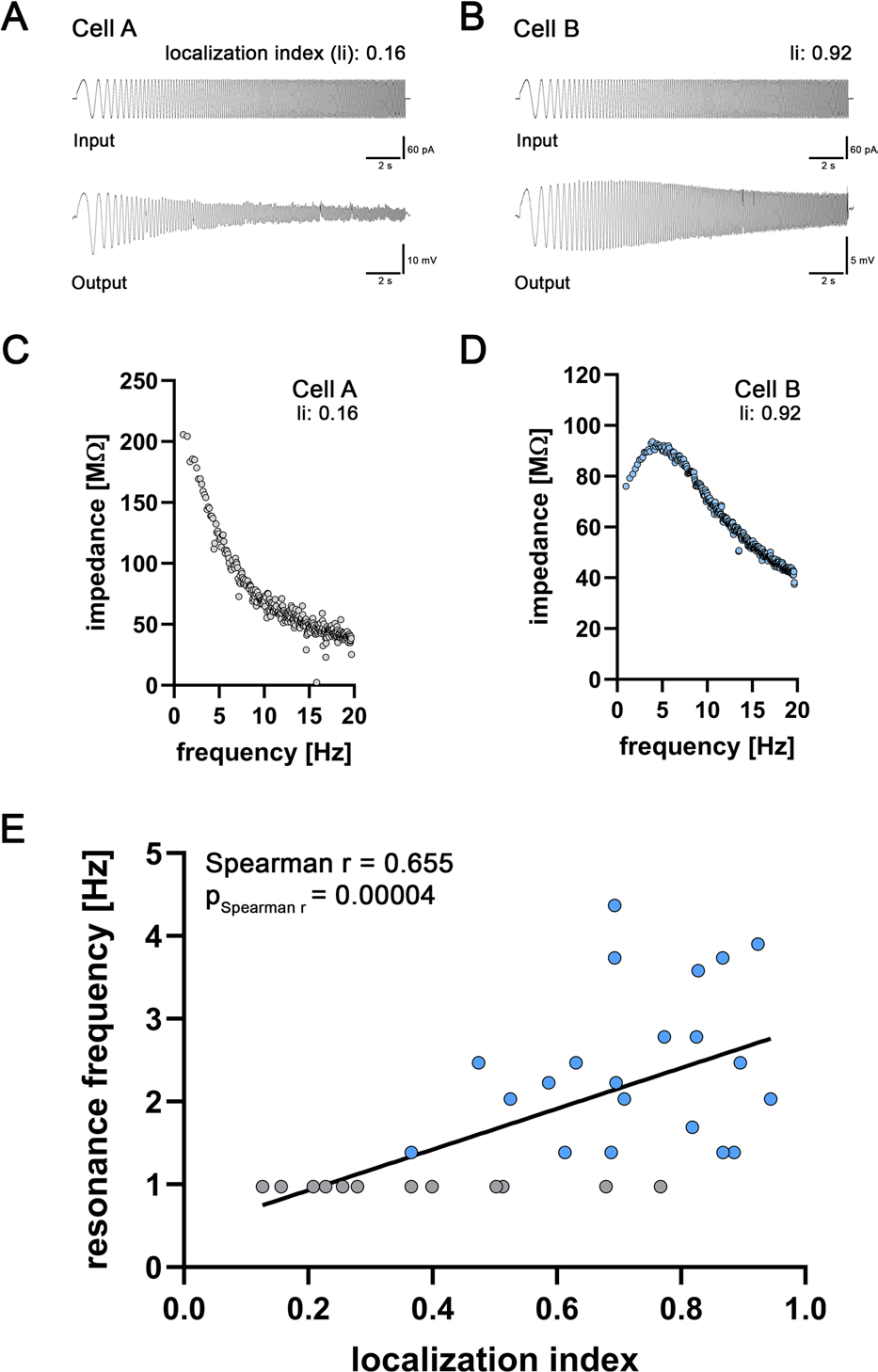
Frequency preference of CA2-PNs dynamically change along the proximodistal axis. (**A**) Sample trace of the subthreshold resonance (lower trace) of a proximally located CA2-PN following a sinusoidal current injection (amplitude: -50 pA to + 50 pA) at 0.97–20 Hz (upper trace). (**B**) Sample trace of the subthreshold resonance (lower trace) of a distally located CA2-PN following a sinusoidal current injection (amplitude: -50 pA to + 50 pA) at 0.97–20 Hz (upper trace). (**C**) Impedance profile of the CA2-PN from (**A**) shows the maximal impedance at the lowest tested frequency. (**D**) Impedance profile of the more distally located CA2-PN from (**B**) shows a detectable peak in the impedance curve at approximately 4 Hz. (**E**) The subthreshold frequency preference of CA2-PNs significantly correlates with the location of the respective neuron along the proximodistal axis. Linear regression is indicated with a black line (n = 33 CA2-PNs; due to action potential generation one CA2-PN was excluded for subthreshold resonance analysis. P-values of Spearman r and Spearman r for correlation analysis are reported in the graphs). Individual values are indicated by colored dots. Cells that demonstrated their maximal impedance at the lowest tested frequency are indicated by a gray dot while the other dots are shown in blue. Source data are provided in Table S5.

Because a hyperpolarization-induced sag is often associated with membrane resonance^19–21^, we assessed resonance features of CA2-PNs along the proximodistal axis (Fig. 5). Impedance calculation was performed based on sinusoidal subthreshold current injections (Fig. 5A, B), and the impedance peak indicated the frequency preference (Fig. 5C, D). Whereas proximal CA2-PNs showed no detectable resonance (grey dots, Fig. 5C), location-dependent resonance emerged in distal CA2-PNs (blue dots, Fig. 5D). We further noted a frequency preference of resonant cells gradually increasing from delta frequency toward the lower theta range (Fig. 5E; cells with resonant properties were indicated by blue dots). We conclude that CA2-PNs *in vitro* exhibit substantial heterogeneity in resonance features, with distal neurons showing a shift toward higher resonance frequencies and a subset reaching the lower theta range.

## Discussion and conclusion

We show that CA2-PNs exhibit pronounced functional heterogeneity along the proximodistal axis despite largely comparable dendritic morphologies *in vitro*. Excitatory synaptic parameters (sEPSCs) did not vary with position, whereas intrinsic membrane properties displayed a gradient with higher input resistance toward CA3 and robust sag and resonance occurring distally toward CA1. Of note, these recordings do not allow assignment of sEPSCs to specific afferent pathways (Schaffer-collateral versus perforant path/entorhinal inputs) and the measured somatic event properties may be influenced by the proximodistal gradient in input resistance, which can shape membrane filtering and the detectability of small-amplitude events. However, somatic whole-cell recordings quantify the effective sEPSC signal arriving at the soma under endogenous membrane properties, and thus capture a functionally relevant readout that may itself contribute to proximodistal heterogeneity or stability in CA2.

Various markers for the identification of CA2 within the hippocampal formation have been established in recent studies. These markers label overlapping but not identical neuronal populations, suggesting heterogeneity of cells and variability in marker-based border definition^7,22^. In this study, we used PCP4 as an established marker for CA2 delineation in the ventral hippocampus^23^. In parallel, connectivity-based definitions include the innervation by Schaffer collaterals and entorhinal input from layer II at distal apical dendrites, which form highly excitable synapses that outperform entorhinal excitatory drive onto CA1-PNs originating from layer III^13^. This observation is consistent with a substantial coupling of CA2 to entorhinal computations. Concordantly, emerging work (e.g., EC→CA2→CA1 disynaptic route^14)^ supports the idea of non-uniform recruitment across CA2. Moreover, recent work in acute/fixed tissue suggests proximodistal morphological differentiation along the CA3-CA2 transition, including variability in thorny excrescences in CA2 subregions, which further implies subregional structural and functional specialization^11^. Although we did not detect gross morphological differences in our data set, the sample size for reconstructions limits sensitivity for subtle or compartment-specific effects. Accordingly, while we did not detect significant position dependence in the measured arbor metrics, we explicitly acknowledge that small, localized, or spine-level differences cannot be excluded.

In this study, we used organotypic entorhino-hippocampal tissue cultures to assess the structural and functional properties of CA2-PNs. These cultures preserve the laminated hippocampal circuit together with cortico-hippocampal projections, enabling measurements in a stabilized neuronal network^24^. In contrast to acute slices, the cultures avoid confounds of acute axotomy and potential dendritic injury during preparation, allowing neuronal properties to be studied under steady-state conditions after structural and synaptic reorganization. An important limitation is, however, that activity-dependent rewiring and culture-specific states can diverge from *in vivo* dynamics, requiring cautious generalization. In this context, the observed proximodistal gradient in intrinsic properties is consistent with prior acute slice and *in vivo* reports and supports the view that key aspects of CA2 organization can be preserved *in vitro*^17^. We conclude that the proximodistal gradient does not depend on immediate fiber tract integrity, but is implemented as an endogenous feature of the CA2 area. Moreover, our results suggest that organotypic tissue cultures are a complementary model to study single-cell and circuit properties of the hippocampal formation.

We observed a gradual change in subthreshold membrane resonance along the CA2 proximodistal axis. This position-dependent tuning supports a resonance sub-organization within CA2 that may not be detected when spatial resolution along the proximodistal axis is not explicitly considered^25^. Resonance and the hyperpolarization-induced sag are commonly attributed to the HCN-mediated hyperpolarization-activated current *I*_h_, which acts as a depolarizing subthreshold conductance that typically lowers input resistance while enabling theta-range band-pass filtering and phase-dependent responsiveness^19,26^. While additional conductance (including potassium currents) may contribute to subthreshold behavior, the co-occurrence of sag and theta-range resonance in our dataset is consistent with a graded contribution of *I*_h_. This interpretation aligns with prior work showing that reducing *I*_h_ increases input resistance and abolishes resonance, and that bidirectional regulation of *I*_h_ tunes input resistance, excitability, and synaptic integration in a frequency-dependent manner^19,26^. Thus, distal CA2-PNs might be biased toward theta oscillation-coupled integration^17^. Notably, a previous study described location-independent spontaneous 3 Hz-oscillations of the membrane potential *V*_m_ in a subset of CA2-PNs that might reflect the influence of local networks^25^. Placed into the broader hippocampal context, these gradients position CA2 between the intrinsic operational features of adjacent subfields. In CA1 pyramidal neurons, HCN-mediated *I*_h_ commonly produces a pronounced sag and supports subthreshold membrane resonance in the theta band, thereby shaping frequency-dependent integration and phase preference during theta-paced network states, particularly during entorhinal theta drive^20,27,28^. In CA3, by contrast, strong recurrent excitation supports fast neuronal dynamics, and CA3 is centrally implicated in gamma oscillations^29^ and the generation and propagation of sharp-wave ripples (SWRs)^30,31^. Together, our observations suggest that CA2 forms a functional transition between these regions: distal CA2-PNs show prominent features reminiscent of CA1-like intrinsic tuning, whereas properties of proximal CA2-PNs might promote efficient recruitment during transient CA3-dominated events.

In this context, previous work revealed that CA2 participates in hippocampal oscillations, including theta, gamma, and sharp-wave ripples (SWRs), with timing control proposed as a key CA2 function^32,33^. Given the essential role of hippocampal oscillations for higher cognitive function, the importance of CA2 in the hippocampal and cortico-hippocampal circuits becomes evident^34,35^. As a general principle in oscillatory networks, membrane resonance (e.g., at theta frequencies) endows neurons with band-pass filtering and phase-locking, biasing synaptic integration and spike timing toward inputs expressed at distinct frequencies^36,37^. The emergence of theta-resonant properties in distal CA2-PNs suggests preferential coupling to entorhinal theta drive and phase-specific recruitment during network states dominated by EC inputs^14,15^. Conversely, non-resonant proximal neurons may be optimized for integration of inputs through mossy fiber synapses and rapid recruitment during transient events, such as SWRs^38,39^. Such differential frequency tuning provides a plausible biophysical substrate for a functional compartmentalization within CA2.

Additionally, CA2 has been implicated in social memory^5,40^, aggression^41,42^, spatial orientation^10,43^, and contributions to episodic memory processing^44,45^, consistent with its connectivity in the hippocampal formation. Moreover, a central role for the CA2 region during epileptogenesis has recently been recognized^46–48^. As a connectivity hub, CA2 can modulate feed-forward inhibition and gate the impact of CA3 and entorhinal inputs on downstream neuronal ensembles^13,14^. While evidence for a differential contribution of proximal and distal CA2-PNs to behavioral control remains limited, circuit-level asymmetries suggest that distal, theta-tuned neurons might preferentially support EC-related computations (e.g., spatial context), whereas proximal neurons may amplify CA3-borne associative content^17,49^. In line with previous work, our findings might therefore inform targeted tests that differentially perturb proximal versus distal CA2 in behaving animals.

In conclusion, we demonstrate that CA2-PNs are not a uniform cell population *in vitro*: intrinsic properties and subthreshold resonance segregate along a proximodistal axis despite comparable dendritic structure and sEPSC properties. This gradient offers a mechanistic basis for input-specific signal integration and phase-dependent participation of CA2 in hippocampal information processing.

## Materials and methods

### Ethics statement

All experimental procedures were performed according to the German animal welfare legislation and approved by the appropriate animal welfare committee and the animal welfare officer at Hannover Medical School (AZ § 4-2023/269). Mice were housed at the Central Animal Laboratories (ZTL, Hannover Medical School) in a 14/10 h light/dark cycle with food and water available *ad libitum*. Experiments involving animals were reported according to ARRIVE guidelines. Every effort was made to minimize distress and pain for animals.

### Preparation of organotypic entorhino-hippocampal tissue cultures

Tissue cultures of the ventral hippocampus and entorhinal cortex were prepared at postnatal day 2–4 from C57BL/6J animals of either sex after decapitation as previously described^50^. Cultivation medium contained 50% (v/v) MEM, 25% (v/v) BME, 25% (v/v) heat-inactivated normal horse serum, 25 mM HEPES buffer solution, 0.15% (w/v) bicarbonate, 0.65% (w/v) glucose, 0.1 mg/ml streptomycin, 100 U/ml penicillin, and 2 mM GlutaMAX. The pH of the medium was adjusted to 7.3 and replaced 3 times per week. All tissue cultures were allowed to mature for at least 18 d in a humidified atmosphere with 5% CO_2_ at 35 °C, and experiments were conducted on cultures aged 20–25 days *in vitro*.

### Whole-cell patch-clamp recordings

Whole-cell patch-clamp recordings in organotypic tissue cultures were performed in a bath solution containing 126 mM NaCl, 2.5 mM KCl, 26 mM NaHCO_3_, 1.25 mM NaH_2_PO_4_, 2 mM CaCl_2_, 2 mM MgCl_2_, and 10 mM glucose in the absence of any channel inhibitors. Recordings were carried out at 35° C under continuous oxygenation (5% CO_2_/95% O_2_) and 2–3 CA2-PNs with deep to middle position within the pyramidal cell band were patched per culture. Cells were visually identified using an LN-Scope (Luigs and Neumann, Germany) equipped with infrared DI-contrast and a 40x water-immersion objective (numerical aperture [NA] 0.8; Olympus). Electrophysiological signals were amplified using a Multiclamp 700B amplifier, digitized with a Digidata 1550B digitizer, and visualized with the pClamp 11 software package. Spontaneous excitatory postsynaptic currents (sEPSCs) of CA2-PNs were recorded in voltage-clamp mode at a holding potential of -70 mV (not corrected for the liquid junction potential). The internal solution contained 126 mM K-gluconate, 4 mM KCl, 10 mM HEPES, 4 mM MgATP, 0.3 mM Na_2_GTP, 10 mM PO-creatine, and 0.2% (w/v) biocytin (pH = 7.25 with KOH; 285 mOsm/kg). Pipettes had a tip resistance of 3–5 MΩ. Series resistance was monitored, and recordings were discarded if the resistance reached ≥ 30 MΩ. The liquid junction potential (LJP) was calculated at 14.5 mV (at 35° C) with the liquid junction potential calculator of the Clampex 11.3 software. The resting membrane potential was *post hoc* corrected for the LJP (V_m_ = V_rec_ – LJP, where V_rec_ is the recorded voltage).

For recording of intrinsic cellular properties in current-clamp mode, pipette capacitance correction was set to 2.0 pF as a standardized value to reduce its influence, and series resistance was compensated using the automated bridge balance tool of the MultiClamp commander. IV-curves were generated by injecting 1 s square pulse currents starting at − 100 pA and increasing in 20 pA steps until + 600 pA current injection was reached (sweep duration: 2 s). To assess neuronal frequency preference, a subthreshold resonance protocol was used in current-clamp mode. Membrane potential of neurons was monitored in response to a sinusoidal current injection (+ 50 pA to -50 pA) with increasing frequency ranging between 0.97 and 20 Hz in 20 s.

### Immunohistochemistry and imaging

For *post hoc* visualization of patched cells, cultures were fixed and stained as previously described^51^. After whole-cell patch-clamp recordings, cultures were fixed for 1 h in 4% paraformaldehyde (PFA; with 4% sucrose) and overnight in 2% PFA (with 30% sucrose). After fixation, cultures were washed in PBS (0.1 M, pH 7.4) and consecutively incubated for 1 h in blocking solution (PBS containing 10% (v/v) NGS and 0.5% (v/v) Triton X-100) to reduce nonspecific staining and to increase tissue penetration. Subsequently, cultures were incubated in staining solution (PBS containing 10% (v/v) NGS and 0.1% (v/v) Triton X-100) with anti-PCP4 antibody (1:1000; #480004 Synaptic Systems^23^ overnight at 4 °C. The following day, cultures were incubated with appropriate AlexaFluor dye-conjugated secondary antibodies (1:1000, goat anti-gp Alexa-488 or -647; #A11073 or #A21450 Invitrogen) and streptavidin AlexaFluor-647 or -488 (1:1000; #S32357 or #S32354 Invitrogen) at 4 °C overnight. DAPI nuclear stain (1:1000 in PBS for 20 min; #62248 Thermo Scientific) was used to visualize cytoarchitecture. Cultures were washed, transferred onto glass slides, and mounted for visualization with DAKO anti-fading mounting medium (#S302380-2 Agilent).

A Leica SP8 laser scanning microscope equipped with a 10× dry (NA 0.4; Leica) and a 20× multi-immersion (NA 0.75; Leica) objective, was used for confocal image acquisition. Images for visualization of PCP4 were acquired with the 10× or the 20× objective (resolution 1024 × 1024). Image stacks for dendritic reconstructions of patched neurons were acquired with a 20× objective at 2× optical zoom (resolution: 512 × 512 pixels; z-step: 2 μm).

### Data and statistics

Electrophysiological data were analyzed using the pClamp 11.3 (Axon Instruments) software. sEPSC properties were analyzed using the automated template search tool for event detection. Input resistance was calculated for the injection of a − 100 pA 1 s square pulse at a time frame of 200 ms at the end of the hyperpolarization (steady-state hyperpolarization). Of note, the steady-state voltage deflection used to compute the input resistance during the − 100 pA step may be influenced by HCN-mediated *I*_h_ activation (i.e., sag), particularly in distal CA2-PNs. Resting membrane potential was calculated as the mean baseline value over all sweeps with *post hoc* correction for the liquid junction potential. To evaluate sag ratio, delta sag peak was defined as the difference between maximal sag peak and steady-state hyperpolarization. Delta sag peak was then divided by the steady-state hyperpolarization to determine sag ratio^20^. To assess rebound ratio, rebound overshoot was divided by the amplitude of hyperpolarization, which was defined as the difference between resting membrane potential and steady-state hyperpolarization. Both sag ratio and rebound ratio were assessed at a current injection of − 100 pA. The membrane time constant was calculated by fitting a predefined standard exponential function via the Chebyshev method in the pClamp 11.3 software to the membrane voltage response at -100 pA current injection for 150 ms after the start of the current injection. To assess action potential frequency and rheobase, the automated threshold search tool of the pClamp 11.3 software was used. For action potential kinetics, the first action potential at rheobase was assessed using the action potential search tool. The resonance frequency was defined as the stimulus frequency at which the sinusoidal current injection evoked the maximal change in membrane potential amplitude. Changes in membrane potential were assessed by using the threshold search tool of pClamp. The impedance amplitude profile was determined by the ratio of the fast Fourier transform (FFT) of the membrane voltage response to the FFT of the sinusoidal current injection. Thus, the resonance frequency depicts the frequency of the sinusoidal current injection where the membrane impedance reaches its maximum value^20^. The lower threshold for theta-band frequencies was defined at 3.5 Hz.

To determine the location of the respective CA2-PN, a localization index was determined. Based on the PCP4 staining, 3 investigators blinded to the location of investigated neurons determined the borders of the CA2 region in regards to CA3 and CA1. Of note, CA2 boundaries can be delineated using complementary criteria, including marker expression (e.g., RGS14, particularly in the dorsal hippocampus), cytoarchitectonic/morphological features, and the position of mossy fiber terminals. These criteria substantially overlap with PCP4-based definitions but may yield slightly different borders, particularly at the CA2-CA1 and CA2-CA3 transitions^22^. Accordingly, boundary assignment remains inherently uncertain and may reflect graded transitions rather than a sharp anatomical cutoff. In the present dataset, we relied on a density-based PCP4 approach with blinded consensus delineation, and used the localization index as a continuous metric to capture proximodistal gradients despite residual boundary uncertainty. The identified border to CA3 was defined as 0.0 while the border to CA1 was defined as 1.0 (Fig. 1B). Neurons outside these borders (thus outside the CA2 region) were excluded for further analysis. The location of each CA2-PN was determined relative to the borders and a numerical value between 0.0 and 1.0 was assigned.

Dendritic morphologies were assessed using the Neurolucida 360 software. Cells were semiautomatically reconstructed with the “user guided tree reconstruction” function. Dendrites were assigned as basal or apical dendrites based on their anatomical localization. In some cases, dendrites originated from the middle part of the soma and projected into the pyramidal cell layer. They were therefore defined as lateral dendrites. Reconstructions were saved as .DAT files, and analysis was performed in the Neurolucida Explorer.

Data were statistically evaluated using GraphPad Prism 10 (GraphPad Software, USA). The correlation matrix for continuous sample data and corresponding p-value calculations were performed with the Spearman r correlation and were depicted as a heatmap. Linear regression was assessed in the XY-graphs and Spearman r and p_Spearman r_ were reported. P-values < 0.05 were considered statistically significant.

### Digital illustrations

Confocal images were stored as .tif files, and image brightness and contrast were adjusted. Figures were prepared using the ImageJ software package (https://imagej.net) and Photoshop graphics software (Adobe, San Jose, CA, USA).

## Supplementary Information

Below is the link to the electronic supplementary material.

Supplementary Material 1

Supplementary Material 2

Supplementary Material 3

Supplementary Material 4

Supplementary Material 5

## Data Availability

Source data and statistical assessments for each figure are provided as supplementary tables. Original data are available from the corresponding authors upon reasonable request.
